# Genetic Polymorphisms in Enzymes Involved in One-Carbon Metabolism and Anti-epileptic Drug Monotherapy on Homocysteine Metabolism in Patients With Epilepsy

**DOI:** 10.3389/fneur.2021.683275

**Published:** 2021-06-09

**Authors:** Shaofang Zhu, Guanzhong Ni, Lisen Sui, Yiran Zhao, Xiaoxu Zhang, Qilin Dai, Aohan Chen, Wanrong Lin, Yinchao Li, Min Huang, Liemin Zhou

**Affiliations:** ^1^Department of Neurology, The First Affiliated Hospital, Sun Yat-sen University, Guangzhou, China; ^2^Department of Epilepsy Center, The Second Affiliated Hospital, Guangzhou University of Chinese Medicine, Guangzhou, China; ^3^Department of Neurology, The Seventh Affiliated Hospital, Sun Yat-sen University, Shenzhen, China; ^4^Laboratory of Drug Metabolism and Pharmacokinetics, School of Pharmaceutical Sciences, Sun Yat-Sen University, Guangzhou, China

**Keywords:** epilepsy, single nucleotide polymorphism, anti-epileptic drug, homocysteine, one-carbon metabolism

## Abstract

**Aims:** To investigate the effects of single nucleotide polymorphisms (SNPs) in genes of one-carbon metabolism (OCM) related enzymes and anti-epileptic drug (AED) monotherapy on homocysteine (Hcy) metabolism in patients with epilepsy, and to further explore specific SNPs that may increase patients' susceptibility to the effects of AEDs on the Hcy imbalance.

**Method:** This case-control study analyzed 279 patients with epilepsy, including patients receiving monotherapy with valproate (VPA) (*n* = 53), oxcarbazepine (OXC) (*n* = 71), lamotrigine (LTG) (*n* = 55), or levetiracetam (LEV) (*n* = 35) and patients who had not taken any AEDs (controls, *n* = 65) for at least 6 months. Serum levels of vitamin B12 (vit B12), folate (FA) and Hcy were measured, and 23 SNPs in 13 genes of OCM-related enzymes were genotyped in all patients.

**Results:** Methylenetetrahydrofolate reductase (*MTHFR*) rs1801133 was associated with elevated serum Hcy levels in patients with epilepsy (*P* < 0.001), and patients presenting the TT genotype exhibited higher serum Hcy levels than patients with the CC (*P* < 0.001) or CT (*P* < 0.001) genotype. A subsequent multiple linear regression analysis showed that AED monotherapy with VPA (vs. control: *P* = 0.023) or OXC (vs. control: *P* = 0.041), and genotypes of *MTHFR* rs1801133 TT (vs. CC: *P* < 0.001; vs. CT: *P* < 0.001), transcobalamin 2 (*TCN2*) rs1801198 CC (vs. GC: *P* = 0.039) and folate receptor 1 (*FOLR1*) rs2071010 AA (vs. GA: *P* = 0.031) were independent risk factors for higher Hcy levels. In the subgroup analysis of patients taking OXC, we found that patients with genotypes of *MTHFR* rs1801133 TT (vs. CC: *P* = 0.001; vs. CT: *P* < 0.001) and *TCN2* rs1801198 CC (vs. GC: *P* = 0.021; vs. GG: *P* = 0.018) exhibited higher serum Hcy levels.

**Conclusions:** VPA, OXC, and genotypes of *MTHFR* rs1801133 TT, *TCN2* rs1801198 CC, and *FOLR1* rs2071010 AA are all independent risk factors for elevated Hcy levels in patients with epilepsy. Moreover, genotypes of *MTHFR* rs1801133 TT and *TCN2* rs1801198 CC may increase patients' susceptibility to the effect of OXC on disrupting Hcy homeostasis.

## Introduction

Previously published data show that patients with epilepsy on chronic anti-epileptic drug (AED) therapy are more susceptible to hyperhomocysteinemia than the general population (1–4). Hyperhomocysteinemia is a dominant probable risk factor for various medical conditions, such as cardiovascular disease (5, 6), osteoporosis (7–9), stroke (10–12), neurodegenerative diseases (13, 14) and neural tube defects (NTDs) (15–17). Moreover, hyperhomocysteinemia may enhance seizure activity and lead to antiepileptic drug resistance as shown in animal model experiments (18–20).

Two pathways are available for the removal of homocysteine (Hcy): transsulfuration and remethylation. In the former pathway, Hcy is catalyzed by cystathionine synthase (CBS) in the presence of serine to form cystathionine, a vitamin B6–dependent reaction. In the latter pathway, both 5-methyltetrahydrofolate (5-mTHF) and betaine can act as methyl donors for the remethylation of Hcy through folate- and betaine-dependent pathways, respectively (21). 5,10-methylenetetrahydrofolate reductase (MTHFR), a key regulatory enzyme, plays an important role in Hcy homeostasis by catalyzing the conversion of 5,10-methylenetetrahydrofolate (5,10-CH_2_-THF) to 5-mTHF, which is catalyzed by methionine synthesis using vitamin B12 as a cofactor for the remethylation of Hcy to methionine (22). Betaine homocysteine methyltransferase (BHMT), which is expressed at high levels in the human liver, also helps maintain the Hcy balance by catalyzing the transfer of a methyl group from betaine to Hcy to generate methionine (23). Then methionine is activated through the action of methionine adenosyltransferase (MAT) to produce s-adenosylmethionine (SAM) which is the ubiquitous methyl donor in a vast array of intracellular transmethylation reactions. Afterwards, s-adenosylhomocysteine (SAH), the end product of all SAM-dependent transmethylation reactions, is rapidly metabolized by SAH hydrolase to produce homocysteine (24). This pathway is the only one that produces Hcy.

As vitamin B12 and folate are essential cofactors for the remethylation of Hcy, decreased blood levels of these nutrients disrupt Hcy metabolism and lead to hyperhomocysteinemia (25). AEDs are believed to interfere with Hcy homeostasis, at least in part, by disturbing the intestinal absorption of folate (FA), influencing CYP450 enzymatic reactions and the subsequent consumption of FA, or changing one-carbon metabolism (OCM)-related enzyme activity (1, 26–28). Phenytoin and carbamazepine are mostly likely to increase homocysteine levels. In recent years, phenytoin is rarely used as monotherapy in the treatment of epilepsy, and carbamazepine has to some extent been replaced by oxcarbazepine in clinical practice (29). However, effects of the second generation AEDs on Hcy metabolism are still waiting to be clarified. Thus, four most commonly used antiepileptic drugs in monotherapy [e.g., valproate (VPA), oxcarbazepine (OXC), lamotrigine (LTG), and levetiracetam (LEV)] were finally included in this study. In addition to the aforementioned environmental factors, genetic factors may also disturb Hcy homeostasis. Single nucleotide polymorphisms (SNPs) in genes involved in the OCM pathway increase blood Hcy levels by changing enzyme activity. The genotypes of *MTHFR* rs1801133 TT and transcobalamin 2 (*TCN2*) rs1801198 GG were reported to be associated with higher Hcy levels (27, 30, 31). A genetic polymorphism in methylenetetrahydrofolate dehydrogenase 1 (*MTHFD1*) rs2236225 was also discovered to increase Hcy levels (32).

However, to date, the effects of genetic polymorphisms in the OCM pathway on blood Hcy levels in patients with epilepsy receiving the most commonly used AED monotherapy are unclear. Thus, we conducted this case-control study to investigate the effects of SNPs in genes encoding OCM-related enzymes and AEDs (e.g., VPA, OXC, LTG or LEV monotherapy) on Hcy levels in patients with epilepsy and to further explore specific SNPs that may increase patients' susceptibility to the effects of AEDs on Hcy levels.

## Materials and Methods

### Subjects

From May 2013 to October 2019, patients with epilepsy (aged between 15 and 55 years) who were treated with VPA (*n* = 53), OXC (*n* = 71), LTG (*n* = 55), or LEV (*n* = 35) monotherapy for at least 6 months, were included in this study. Patients with epilepsy who were not treated with any AED for at least 6 months were enrolled as controls (*n* = 65). Epilepsy caused by ischemic stroke or coexisting with cardiac or peripheral vascular diseases, hematological diseases, tumors, liver or renal diseases constituted criteria resulting in exclusion from the study. All subjects who regularly consumed vitamins or any other drugs, other than AEDs (i.e., levodopa, fibrates, niacin, statins, metformin, methotrexate, sulfasalazine, and so on), known to affect plasma levels of FA or Hcy were also excluded. Patients in the two groups were from the same geographic area and were matched for age, sex and ethnic background. The current study was approved by the Human Ethics Committee of the First Affiliated Hospital, Sun Yat-sen University, and written informed consent was obtained from each participant.

### Biochemical Analyses

Approximately 2 ml of venous blood was collected from participants in the sitting position. Serum was quickly separated by centrifugation and stored at −80°C until assayed. The serum levels of vitamin B12, FA and Hcy were detected using an Immulite 2000 autoanalyzer and suitable kits (DPC Diagnostic Products Corporation, Los Angeles, CA, USA) according to the manufacturer's instructions.

### Genetic Analyses

Whole-genome DNA was extracted from peripheral blood cells using a TIANamp Blood DNA Kit (Qiagen, Beijing, China) according to the manufacturer's instructions. The DNA concentration and purity were determined by measuring the absorbance at 260 and 280 nm using a NanoDrop^TM^ 1,000 Spectrophotometer (Thermo Scientific, Wilmington, USA). Twenty-three SNPs in 13 genes of the OCM pathway were detected by an iPLEX® mass spectrometry-based multiplex genotyping assay (Sequenom, CA, USA), including *GCPII* rs202676, *FOLR1* rs2071010, *FOLR2* rs2298444, *SLC*19A1 rs1051266/rs914238, *DHFR* rs380691, *MTHFD1* rs1950902/rs2236225, *MTHFR* rs1801131/rs1801133, *TCN2* rs1801198, *MTRR* rs1801394, *BHMT* rs3733890, *DNMT1* rs2114724/rs2241531/rs7253062, *DNMT3a* rs13036246/rs34048824/rs6722613/rs7575625/rs7587636, and *DNMT3b* rs2424908/rs6141813, according to a previously described method (33). In Supplementary Material listed 23 candidate SNPs and its PCR primer and extension primer. MassARRAY Typer 4.0 software was used for proper data acquisition and analysis. Assays with a <80% call rate within the same SpectroCHIP were considered as having failed.

### Statistical Analysis

SPSS 20.0 for Windows and Prism 8.0.1 were used for statistical analyses. Analyses of parametric variables were performed using either student's *t*-test or one-way analysis of variance (ANOVA) with a *post hoc* Bonferroni test, and the results are described as the means ± standard deviations (x ± s). In analyses of non-parametric variables, either the Mann-Whitney *U*-test or Kruskal-Wallis test with *post hoc* Mann-Whitney *U*-test was used. Furthermore, a multiple linear regression analysis was employed to analyze the factors influencing serum Hcy levels. A *P*-value < 0.05 was considered statistically significant.

## Results

### Demographic Data

All patients in treatment groups had daily dosages within therapeutic range. Table 1 shows the main characteristics of the study population. No differences in age or sex distribution were observed among the groups. The serum levels of FA and vitamin B12 were significantly different among the five groups (*P* = 0.004 and *P* < 0.001, respectively) (Table 1).

**Table 1:** Demographic features of the subjects.

	**Control**	**VPA**	**OXC**	**LTG**	**LEV**	***P*-value**
*N*	65 (M: 31)	53 (M: 31)	71 (M: 35)	55 (M: 19)	35 (M: 17)	0.173
Age, Y	28.58 ± 10	25.81 ± 10.15	26.15 ± 8.84	27.35 ± 6.57	26.26 ± 8.67	0.426
FA, nmol/l	23.38 ± 9.95	28.2 ± 12.1	21.09 ± 8.18	25.86 ± 11.74	23.15 ± 11.59	0.004
vit B12, pmol/l	368.82 ± 169.9	460.47 ± 185.85	307.93 ± 134.65	361.84 ± 143.62	327.75 ± 146.63	<0.001
Hcy, μmol/l	12.06 ± 3.55	12.84 ± 5.59	14.86 ± 8.35	12.42 ± 5.4	14.14 ± 7.13	0.063

*Data are presented as means ± SD. The mean serum levels of FA, vit B12 and Hcy among groups were compared using ANOVA. P < 0.05 was considered statistically significant*.

*VPA, valproate; OXC, oxcarbazepine; LTG, lamotrigine; LEV, levetiracetam; N, number; FA, folate; vit B12, vitamin B12; Hcy, homocysteine*.

**Figure 1:**
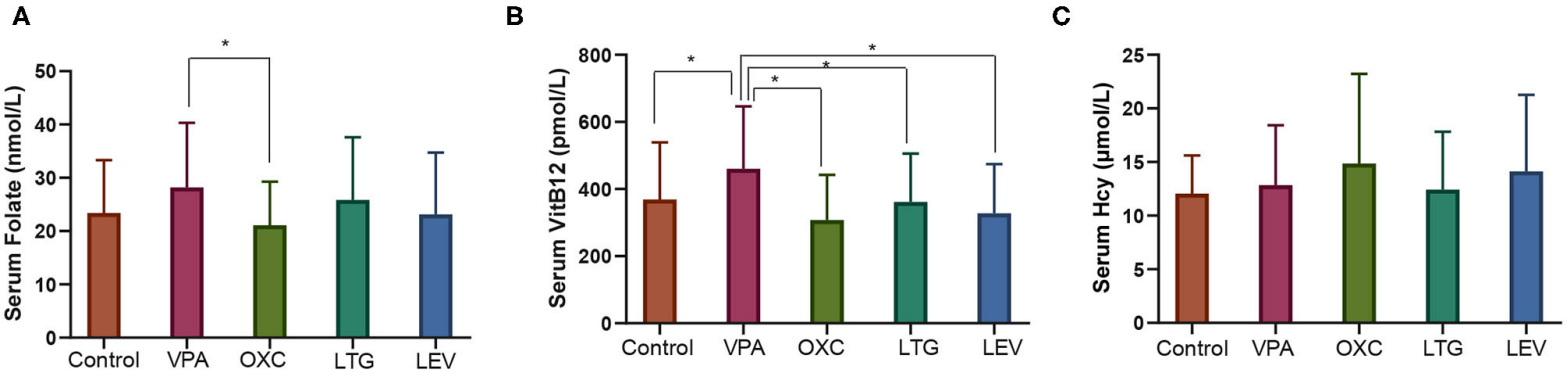
Serum levels of one-carbon metabolites among groups of patients with epilepsy. **(A)** Serum FA levels, **(B)** serum VitB12 levels, and **(C)** serum Hcy levels. *P < 0.05. VPA, valproate; OXC, oxcarbazepine; LTG, lamotrigine; LEV, levetiracetam; *N*, number; vit B12, vitamin B12; Hcy, homocysteine.

Patients on VPA monotherapy exhibited higher FA levels than patients on OXC monotherapy (28.2 ± 12.1 vs. 21.09 ± 8.18 nmol/l; *P* = 0.003) (Figure 1A) and higher vitamin B12 levels than patients in the non-AED (460.47 ± 185.85 vs. 368.82 ± 169.9 pmol/l; *P* = 0.018), LTG (vs. 361.84 ± 143.62 pmol/l; *P* = 0.012), LEV (vs. 327.75 ± 146.63 pmol/l; *P* = 0.001) and OXC monotherapy groups (vs. 307.93 ± 134.65 pmol/l; *P* < 0.001) (Figure 1B). However, the differences in serum Hcy levels among groups were not statistically significant (*P* > 0.05) (Figure 1C).

### Effects of SNPs in Genes Encoding OCM-Related Enzymes on Hcy Levels in Patients With Epilepsy

The associations between genetic polymorphisms in OCM-related enzymes and serum levels of Hcy in patients with epilepsy were analyzed. In this study, we observed that *MTHFR* rs1801133 was associated with serum Hcy levels in patients with epilepsy (CC vs. CT vs. TT: 12.4 ± 5.01 vs. 13.44 ± 4.79 vs. 19.92 ± 15.39 μmol/l, *P* < 0.001), with the TT genotype resulting in higher serum Hcy levels than the CC (*P* < 0.001) or CT (*P* < 0.001) genotype (Table 2).

**Table 2:** Effects of SNPs in genes encoding OCM-related enzymes on Hcy levels in patients with epilepsy.

**Gene**	**SNPs**	**Serum Hcy (μmol/l)**			***P*-value**
		**wild type**	**Heterozygotes**	**Homozygotes**	
*GCPII*	rs202676	13.18 ± 5.67 (AA: *n* = 137)	13.15 ± 6.79 (AG: *n* = 116)	13.17 ± 4.52 (GG: *n* = 24)	0.999
*FOLR1*	rs2071010	13.69 ± 7.13 (GG: *n* = 188)	12.1 ± 3.47 (GA: *n* = 83)	15.04 ± 6.42 (AA: *n* = 8)	0.112
*FOLR2*	rs2298444	13.91 ± 7.46 (TT: *n* = 105)	12.93 ± 5.80 (TC: *n* = 129)	12.67 ± 4.26 (CC: *n* = 45)	0.392
*SLC19A1*	rs1051266	13.06 ± 5.67 (TT: *n* = 80)	13.53 ± 5.69 (TC: *n* = 129)	12.7 ± 8.18 (CC: *n* = 62)	0.676
	rs914238	13.13 ± 6.73 (TT: *n* = 89)	13.55 ± 6.26 (TC: *n* = 137)	12.64 ± 5.62 (CC: *n* = 51)	0.665
*DHFR*	rs380691	13.26 ± 6.22 (GG: *n* = 99)	13.48 ± 6.53 (GA: *n* = 127)	12.75 ± 5.95 (AA: *n* = 51)	0.786
*MTHFD1*	rs1950902	13.17 ± 6.89 (GG: *n* = 115)	13.36 ± 5.72 (GA: *n* = 125)	13.12 ± 6.36 (AA: *n* = 38)	0.965
	rs2236225	13.26 ± 6.85 (GG: *n* = 166)	13.22 ± 5.31 (GA: *n* = 100)	13.24 ± 6.5 (AA: *n* = 10)	0.999
*MTHFR*	rs1801131	13.75 ± 6.87 (TT: *n* = 159)	12.88 ± 5.6 (TG: *n* = 101)	11.48 ± 3.94 (GG: *n* = 15)	0.289
	rs1801133	12.4 ± 5.01 (CC: *n* = 156)^b^	13.44 ± 4.79 (CT: *n* = 106)^c^	19.92 ± 15.39 (TT: *n* = 17)	<0.001^a^
*TCN2*	rs1801198	13.37 ± 6.83 (GG: *n* = 95)	12.63 ± 4.21 (GC: *n* = 131)	14.53 ± 8.97 (CC: *n* = 52)	0.177
*MTRR*	rs1801394	12.88 ± 6.4 (AA: *n* = 147)	13.63 ± 6.21 (AG: *n* = 103)	14.62 ± 7.05 (GG: *n* = 19)	0.425
*BHMT*	rs3733890	12.59 ± 6.21 (GG: *n* = 131)	13.78 ± 6.61 (GA: *n* = 118)	13.80 ± 4.81 (AA: *n* = 26)	0.29
*DNMT1*	rs2114724	13.6 ± 6.49 (CC: *n* = 143)	12.93 ± 6.49 (CT: *n* = 107)	12.65 ± 4.17 (TT: *n* = 28)	0.615
	rs2241531	12.92 ± 6.22 (CC: *n* = 83)	12.87 ± 5.18 (CG: *n* = 139)	14.79 ± 8.52 (GG: *n* = 55)	0.134
	rs7253062	13.67 ± 6.77 (GG: *n* = 143)	12.84 ± 5.84 (GA: *n* = 119)	13.2 ± 5.21 (AA: *n* = 14)	0.576
*DNMT3a*	rs13036246	13 ± 5.44 (CC: *n* = 143)	14.02 ± 7.54 (CT: *n* = 110)	11.5 ± 4.12 (TT: *n* = 25)	0.149
	rs34048824	13.07 ± 5.65 (TT: *n* = 168)	13.83 ± 7.46 (TC: *n* = 97)	11.54 ± 4.1 (CC: *n* = 14)	0.368
	rs6722613	13.14 ± 5.71 (GG: *n* = 113)	13.51 ± 6.8 (GA: *n* = 128)	12.79 ± 6.24 (AA: *n* = 37)	0.796
	rs7575625	13.12 ± 5.72 (AA: *n* = 160)	13.6 ± 7.25 (AG: *n* = 106)	12.09 ± 4.02 (GG: *n* = 13)	0.662
	rs7587636	13.14 ± 5.78 (GG: *n* = 119)	13.62 ± 7.02 (GA: *n* = 133)	11.98 ± 4.16 (AA: *n* = 27)	0.452
*DNMT3b*	rs2424908	12.88 ± 4.83 (TT: *n* = 96)	13.45 ± 6.61 (TC: *n* = 138)	13.46 ± 7.89 (CC: *n* = 45)	0.776
	rs6141813	13.17 ± 5.7 (AA: *n* = 116)	13.15 ± 5.99 (AG: *n* = 130)	14.11 ± 9.07 (GG: *n* = 32)	0.722

a *Serum Hcy levels were significantly different among groups*.

b *TT vs. CC: 19.92 ± 15.39 vs. 12.4 ± 5.01 μmol/l, P < 0.001*.

c *TT vs. CT: 19.92 ± 15.39 vs. 13.44 ± 4.79 μmol/l, P < 0.001*.

*P-values were calculated using ANOVA*.

*SNPs, some single nucleotide polymorphisms; Hcy, homocysteine*.

### Effects of AEDs and SNPs in OCM Related Enzymes on Serum Hcy Levels in Patients With Epilepsy

We employed multiple linear regression analysis to further explore the effects of AEDs and SNPs in OCM-related enzymes on serum Hcy levels. After adjusting for other factors included in the model, the analysis showed that serum levels of FA and vitamin B12 were negatively associated with serum Hcy levels (FA: β = −0.192, *P* < 0.001; VitB12: β = −0.008, *P* < 0.001, respectively). It also showed that monotherapy with VPA (vs. Control: β = 2.406, *P* = 0.023) or OXC (vs. Control: β = 1.968, *P* = 0.041) and genotypes of *MTHFR* rs1801133 TT (vs. CC: β = 6.334, *P* < 0.001; vs. CT: β = 6.516, *P* < 0.001), *TCN2* rs1801198 CC (vs. GC: β = 1.91, *P* = 0.039) and folate receptor 1 (*FOLR1*) rs2071010 AA (vs. GA: β = 4.464, *P* = 0.031) were independent risk factors for higher Hcy levels. According to the standard partial regression coefficient, the *MTHFR* rs1801133 TT genotype had the greatest effect on Hcy levels among these factors, and VPA had a stronger effect on Hcy levels than OXC (Table 3).

**Table 3:** Effects of AEDs, SNPs and vitamins on Hcy levels in patients with epilepsy.

**Model**	**Unstandardized coefficients**		**Standardized coefficients**	***P*-value**	**VIF**	***R*^**2**^**
	**β**	**Std. error**	**Beta**			
(Constant)	17.645	1.465		<0.001		0.28
FA (nmol/l)	−0.192	0.032	−0.33	<0.001	1.125	
vit B12 (pmol/l)	−0.008	0.002	−0.205	<0.001	1.156	
VPA	2.406	1.048	0.151	0.023	1.587	
OXC	1.968	0.957	0.137	0.041	1.628	
LTG	0.435	1.011	0.028	0.668	1.519	
LEV	1.421	1.157	0.075	0.22	1.379	
rs1801133 CT	−0.182	0.719	−0.014	0.801	1.141	
rs1801133 TT	6.334	1.424	0.242	<0.001	1.09	
rs1801198 CC	1.91	0.92	0.119	0.039	1.204	
rs1801198 GG	0.716	0.745	0.054	0.337	1.168	
rs2071010 AA	4.464	2.063	0.119	0.031	1.113	
rs2071010 GG	1.207	0.727	0.09	0.098	1.089	

*The overall significance for Hcy levels was F = 8.583; P < 0.001. P < 0.05 was considered a statistically significant difference*.

*FA, folate; vit B12, vitamin B12; VPA, valproate; OXC, oxcarbazepine; LTG, lamotrigine; LEV, levetiracetam; VIF, variable inflation factor*.

In the subgroup analysis of patients taking OXC, we found that genotypes of *MTHFR* rs1801133 TT (vs. CC: β = 13.282, *P* = 0.001; vs. CT: β = 14.814, *P* < 0.001) and *TCN2* rs1801198 CC (vs. GC: β = 5.432, *P* = 0.021; vs. GG: β = 5.905, *P* = 0.018) resulted in higher serum Hcy levels (as shown in Table 4). However, similar relationships were not observed for patients on VPA. We suspected that *MTHFR* rs1801133 TT and *TCN2* rs1801198 CC genotypes may make patients susceptible to the effect of OXC on increasing Hcy levels.

**Table 4:** Effects of SNPs and vitamins on Hcy levels in patients with epilepsy receiving OXC monotherapy.

**Model**	**Unstandardized coefficients**		**Standardized coefficients**	***P*-value**	**VIF**	***R*^**2**^**
	**β**	**Std. error**	**Beta**			
(Constant)	21.058	2.895		<0.001		0.378
rs1801133 CT	−1.534	1.746	−0.092	0.383	1.141	
rs1801133 TT	13.282	3.729	0.370	0.001	1.124	
rs1801198 CC	5.432	2.297	0.246	0.021	1.127	
rs1801198 GG	−0.473	1.838	−0.027	0.798	1.125	
FA (nmol/l)	−0.334	0.106	−0.327	0.002	1.119	

*The overall significance for Hcy levels was F = 7.891; P < 0.001. P < 0.05 was considered a statistically significant difference*.

*FA, folate; VIF, variable inflation factor*.

## Discussion

The first generation enzyme-inducing antiepileptic drugs, such as phenytoin and carbamazepine, may cause a deficiency of folate by influencing the activity of the hepatic enzymes and hence increase Hcy levels (1, 28). Compared with them, the second generation AEDs are less likely to stimulate enzymes of the liver and then are supposed less likely to disrupt Hcy metabolism, however, the conclusions are still waiting to be drawn. In this study, phenytoin and carbamazepine were excluded, because none of the patients took phenytoin monotherapy and only four patients took carbamazepine monotherapy in our clinic practice, while VPA, OXC, LTG and LEV, the most commonly used antiepileptic drugs in monotherapy, were included in this study.

In our study, after adjusting for related risk factors, such as FA levels, vitamin B12 levels and some OCM-related enzyme SNPs, LTG and LEV monotherapy were innocent of increasing Hcy levels, while OXC and VPA were associated with increased Hcy levels in patients with epilepsy. Several published studies found that in patients stabilized on LTG and LEV monotherapy, blood Hcy level were not significantly different from those observerd in controls (28, 34), similar to our findings. Conversely, a prospective longitudinal study showed that 6 months of LEV and OXC monotherapy significantly increased Hcy levels in patients with newly diagnosed epilepsy who were drug-free at baseline (35). Another study found that OXC therapy was associated with increased Hcy levels, even after controlling for sex, age, vitamin B12 levels, FA levels and the *MTHFR* rs1801133 TT genotype (28). Regarding the effect of VPA on Hcy levels, previous work by our research members, including a meta-analysis and a previously published study, also suggested that VPA was associated with high Hcy levels (27, 36). However, a small-sample study showed that VPA had no effect on Hcy levels in children with epilepsy compared with healthy children (26). Thus, although AEDs, including OXC, are generally recognized to increase Hcy levels by interfering with important cofactors (e.g., FA and vitamin B12) in the OCM pathway (37), the effect of VPA on Hcy levels and the underlying mechanism have still not been completely clarified. As shown in this study, treatment with VPA monotherapy was associated with higher vitB12 levels than treatment with LTG, LEV, OXC or controls. The existing literature also shows that VPA-related increases in Hcy levels may not be reduced by FA and vitamin B12 supplementation within a certain range (1). According to Anna et al., vitamin supplementation, including folate (0.4 mg a day), magnesium with 50 mg of vitamin B6 and vitamin B12 (100 μg a day), in 23 VPA-treated patients with chronic epilepsy for 1 year significantly increases s-FA levels (before vs. after supplementation: 8.4 ± 4.2 vs. 9.7 ± 4.5 ng/ml, *P* = 0.04), but p-tHcy levels are not decreased (before vs. after supplementation: 9.8 ± 3.4 vs. 9.3 ± 1.4 μmol/l, *P* > 0.05) (1). Taken together, we speculate that the increased Hcy levels caused by AEDs are not entirely dependent on deficiencies in FA and vitamin B12 but also dependent on additional mechanisms that remain to be elucidated.

SNPs in OCM-related enzymes may also be involved in disturbing OCM by changing enzyme activity. MTHFR is the critical enzyme that catalyzes the transformation of 5,10-CH_2_-THF to 5-mTHF. 5-mTHF, the major circulating form of folate, acts as a C donor for the vitamin B12-dependent remethylation of Hcy to methionine (38). The *MTHFR* rs1801133 TT genotype is significantly associated with decreased MTHFR specific activity (38); therefore, it might increase Hcy levels and serve as a risk factor for congenital malformations, such as NTDs and cleft lip with or without cleft palate (CL/P) (30). In our study, serum Hcy levels in patients with the *MTHFR* rs1801133 TT genotype were significantly higher than in patients with the *MTHFR* rs1801133 CT or CC genotype, even after adjusting for multiple related risk factors. The *MTHFR* rs1801133 TT genotype was also shown to be an independent risk factor for increased blood Hcy levels in patients with epilepsy taking OXC monotherapy.

TCN2, a cobalamin-transporting protein, mediates the transmembrane transport of cobalamin, which is a key cofactor in the reaction catalyzing the methylation of Hcy to methionine (39). The clinical importance of polymorphisms in *TCN2* rs1801198 remains controversial. A systematic review and meta-analysis showed no significant association between *TCN2* rs1801198 and FA levels or primary risks of congenital abnormalities; however, in individuals of European descent, Hcy levels were significantly higher in subjects with the *TCN2* rs1801198 GG genotype than in subjects with the *TCN2* rs1801198 CC genotype (31). A family-based, candidate gene association study of non-syndromic cleft palate only (CPO), which included 129 Italian and 65 Asian families, found no evidence of an association between *TCN2* rs1801198 and CPO (40). However, another study reported that the *TCN2* rs1801198 GG genotype was associated with an increased risk of fetal cleft lip with or without cleft palate in Californian women with low folate intake, although the sample size was too small to obtain meaningful conclusions (41). In addition, Martinelli et al. also identified a causative role for the *TCN2* rs1801198 GG genotype in CL/P in Italy (42); however, in another recently published study, the same research group reported that the *TCN2* rs1801198 GG genotype was associated with a decreased risk of cleft in Iraqi children (43). In this study, we observed that the *TCN2* rs1801198 CC genotype was an independent risk factor for higher Hcy levels not only in all patients with epilepsy (CC vs. GC: β = 1.91, *P* = 0.039) but also specifically in patients on OXC (CC vs. GC: β = 5.432, *P* = 0.021; CC vs. GG: β = 5.905, *P* = 0.018). Consequently, the effect of genetic polymorphisms in *TCN2* rs1801198 on blood Hcy levels requires further studied.

FOLR1 is a high-affinity folate receptor that transports folate, preferably the oxidized form of folate, via receptor-mediated endocytosis (44). A recent study in India including 206 probands with autism spectrum disorder (ASD) and 286 age-matched controls revealed a higher occurrence of the *FOLR1* rs2071010 AA genotype in the probands with ASD, more specifically in the male subjects, compared with gender-matched controls (*P* = 0.02; CI 1.28–32.64), thereby indicating a positive association of the *FOLR1* rs2071010 AA genotype with ASD (45). Our study showed that *FOLR1* rs2071010 AA genotype was an independent risk factor for higher Hcy levels.

Although a single key genetic factor may disturb Hcy homeostasis, gene-gene or gene-environment interactions may also be involved in Hcy metabolism. Subsequently, one of the limitations of this paper is that we only included 23 SNPs in the OCM pathway in this study, which is not an extensive list. Second, other environmental factors that might affect the results, such as the intake of dietary folate, were not excluded in this study. Third, although we choose patients with epilepsy not taking AEDs for at least 6 months as controls, which might help to exclude the effects of being epileptic on Hcy metabolism in our subject, however, lack of healthy controls may make our study less rigorous. Finally, the small sample size in this study was also a limitation and larger sample studies are warranted to validate our findings.

## Conclusion

We investigated the effects of 23 SNPs in 13 genes encoding OCM-related enzymes and AED monotherapy (e.g., VPA, OXC, LTG or LEV) on blood Hcy levels in patients with epilepsy. Based on our results, VPA, OXC, and genotypes of *MTHFR* rs1801133 TT, *TCN2* rs1801198 CC and *FOLR1* rs2071010 AA are all independent risk factors for elevated Hcy levels. The *MTHFR* rs1801133 TT and *TCN2* rs1801198 CC may be susceptible genotypes to increase blood Hcy levels in patients with epilepsy, especially when combined with OXC monotherapy. Thus, genotyping those three SNPs in patients with epilepsy, especially those who are taking OXC, might be of a certain significance in guiding clinical medications.

## Data Availability Statement

The data presented in the study are deposited in the dbSNP repository: https://www.ncbi.nlm.nih.gov/SNP/snp_viewTable.cgi?handle=FAHOSYSU.

## Ethics Statement

The studies involving human participants were reviewed and approved by Human Ethics Committee of the First Affiliated Hospital, Sun Yat-sen University. Written informed consent to participate in this study was provided by the participants' legal guardian/next of kin.

## Author Contributions

SZ: conceptualization, methodology, investigation, and writing– original draft. GN: methodology, formal analysis, and investigation. LS: resources, data curation, and writing– review and editing. YZ: validation and software. XZ: software and formal analysis. QD: resources and writing– review and editing. AC: software and data curation. WL: data curation. YL: visualization. MH: resources. LZ: writing– review and editing, supervision, project administration, and funding acquisition. All authors contributed to the article and approved the submitted version.

## Conflict of Interest

The authors declare that the research was conducted in the absence of any commercial or financial relationships that could be construed as a potential conflict of interest.

## Abbreviations

SNPs: some single nucleotide polymorphisms
OCM: one-carbon metabolism
AED: antiepileptic drug
Hcy: homocysteine
VPA: valproate
OXC: oxcarbazepine
LTG: lamotrigine
LEV: levetiracetam
vit B12: vitamin B12
FA: folate
MTHFR: methylenetetrahydrofolate reductase
TCN2: transcobalamin 2
FOLR1: folate receptor 1
NTDs: neural tube defects
CBS: cystathionine synthase
5-mTHF: 5-methyltetrahydrofolate
5, 10-CH2-THF, 5: 10-methylenetetrahydrofolate
BHMT: betaine homocysteine methyltransferase
MAT: methionine adenosyltransferase
SAM: s-adenosylmethionine
SAH: s-adenosylhomocysteine
MTHFD1: methylenetetrahydrofolate dehydrogenase 1
ANOVA: one-way analysis of variance
CL/P: cleft lip with or without cleft palate
CPO: nonsyndromic cleft palate only
ASD: autism spectrum disorder.
