# Supplementary material for: Genetic Polymorphisms in Enzymes Involved in One-Carbon Metabolism and Anti-epileptic Drug Monotherapy on Homocysteine Metabolism in Patients With Epilepsy
Source: Front Neurol. 2021 Jun 9;12:683275. doi: 10.3389/fneur.2021.683275 (PMC8220895; doi:10.3389/fneur.2021.683275)
Supplement: Supplementary file 1 [file Table_1.DOC]

| Sequenom MassARRAY PCR primers of 23 SNPs in 13 genes of the OCM pathway | | |
| --- | --- | --- |
| Gene | rs number | Primers |
| *GCPII* | rs202676 | F:ACGTTGGATGGTCCATATAAACTTTCGAGG |
|  |  | R:ACGTTGGATGTGGATGAATTGAAAGCTGAG |
|  |  | E:GGGTTTTCGAGGATGTACTTACT |
| *FOLR1* | rs2071010 | F:ACGTTGGATGCTGAAGGCCAGAGAATCTTG |
|  |  | R:ACGTTGGATGGGGATGAACACTAACTTGTC |
|  |  | E:GACCGAGTGTGGCCTGCTCAAG |
| *FOLR2* | rs2298444 | F:ACGTTGGATGGGAGATTGAGGTAGGGTTTG |
|  |  | R:ACGTTGGATGCAAAGCTCATTTCTGGCCAC |
|  |  | E:GGTAGGGTTTGGAAAATC |
| *SLC19A1* | rs1051266 | F:ACGTTGGATGTGAAGCCGTAGAAGCAAAGG |
|  |  | R:ACGTTGGATGAGAAGCAGGTGCCCGTGGAA |
|  |  | E:GCACCGAGCTCCGGTCCTGGCGGC |
|  | rs914238 | F:ACGTTGGATGTGCCTGGTGTGTGTGTCTTT |
|  |  | R:ACGTTGGATGTACATGCAGACAGACGGACA |
|  |  | E:GCTCACATCTACATGTGCG |
| *DHFR* | rs380691 | F:ACGTTGGATGTCTAAACACGGCTTGAGTTG |
|  |  | R:ACGTTGGATGGGAAAACCCTATGGGTGAAC |
|  |  | E:GGTGGGTTGCTGAGGCTCCTGACT |
| *MTHFD1* | rs1950902 | F:ACGTTGGATGAGATTGACTAGCATCAATG |
|  |  | R:ACGTTGGATGCCTTAGGCGTACAAGGAATG |
|  |  | E:GAGGTCACCTCTAGCAAGT |
|  | rs2236225 | F:ACGTTGGATGACATCGCACATGGCAATTCC |
|  |  | R:ACGTTGGATGTAACCTACAAACCCTTCTGG |
|  |  | E:ATTACTCCATCATTGCAGACC |
| *MTHFR* | rs1801131 | F:ACGTTGGATGTCTCCCGAGAGGTAAAGAAC |
|  |  | R:ACGTTGGATGAGAGCAAGTCCCCCAAGGAG |
|  |  | E:GAGCTGACCAGTGAAG |
|  | rs1801133 | F:ACGTTGGATGGTGCATGCCTTCACAAAGCG |
|  |  | R:ACGTTGGATGCACTTGAAGGAGAAGGTGTC |
|  |  | E:GCGTGATGATGAAATCG |
| *TCN2* | rs1801198 | F:ACGTTGGATGCCTCACTCTATCACCAGTTC |
|  |  | R:ACGTTGGATGAGACATGCTGTTCCCAGTTC |
|  |  | E:TCCCCATGACTTCCCCCATGC |
| *MTRR* | rs1801394 | F:ACGTTGGATGCTATATGCTACACAGCAGGG |
|  |  | R:ACGTTGGATGGAAAATCCATGTACCACAGC |
|  |  | E:GGCAGGTACCACAGCTTGCTCACA |
| *BHMT* | rs3733890 | F:ACGTTGGATGAGTGAAGCTCATGAAGGAGG |
|  |  | R:ACGTTGGATGAGGAGTGTGGTAAGCCAAGG |
|  |  | E:CCTATGGCTTGGAGGCTGCCC |
| *DNMT1* | rs2114724 | F:ACGTTGGATGAGGATGTGGGCCATGCTCTA |
|  |  | R:ACGTTGGATGTCACGCTGGGACAGAGGTAA |
|  |  | E:TTAAAGAGGTAAGGATGCGGC |
|  | rs2241531 | F:ACGTTGGATGCTGAAACCCCTTCCCTTTTG |
|  |  | R:ACGTTGGATGACCCCCAAAGAACCGTAAGA |
|  |  | E:ATTTTAACATTACCATCTGCTTT |
|  | rs7253062 | F:ACGTTGGATGTAGAACTGCCAGGAGTTTGC |
|  |  | R:ACGTTGGATGTGTCCCTGAAAGAGTCAAGC |
|  |  | E:GTCAGTAATCAAATCAGACCA |
| *DNMT3a* | rs13036246 | F:ACGTTGGATGGGAATAAGATAATGCATGCAC |
|  |  | R:ACGTTGGATGTGGCCTCAGAAACACAATGG |
|  |  | E:GCATGCACATAGCACC |
|  | rs34048824 | F:ACGTTGGATGATCTGTAAACTGGGGCCATC |
|  |  | R:ACGTTGGATGGGTTCTATTTGGTCCTTGGG |
|  |  | E:CCCTGCCCCATTTGT |
|  | rs6722613 | F:ACGTTGGATGCCTTTCGTGTTGCTCAATGG |
|  |  | R:ACGTTGGATGCCAGACACATACTGGAGGAT |
|  |  | E:CAGCAGTCCCTCAAAATC |
|  | rs7575625 | F:ACGTTGGATGAGACAATGAAGCCAGGTAGC |
|  |  | R:ACGTTGGATGTGTGCAAAACCACTAGAGGC |
|  |  | E:TTCACAAGGCTACACTT |
|  | rs7587636 | F:ACGTTGGATGAGCTCAAGCTTTGGTTCCAG |
|  |  | R:ACGTTGGATGACAATGTTGCCACATCCACG |
|  |  | E:GGTTCCAGATTTTTAAATTCTCA |
| *DNMT3b* | rs2424908 | F:ACGTTGGATGCTAGGATTCTGCTCCAATGC |
|  |  | R:ACGTTGGATGTCCACTTTGAGAACCCCTTG |
|  |  | E:CCCGGCTGCCCCTCATTCT |
|  | rs6141813 | F:ACGTTGGATGGGACATCTAGCTTAGTGGTG |
|  |  | R:ACGTTGGATGCAGTACCATGAACTGAGAGC |
|  |  | E:TCTCCAGCCAGCTCCC |
